# Activation of the NLRP3 Inflammasome by IAV Virulence Protein PB1-F2 Contributes to Severe Pathophysiology and Disease

**DOI:** 10.1371/journal.ppat.1003392

**Published:** 2013-05-30

**Authors:** Julie L. McAuley, Michelle D. Tate, Charley J. MacKenzie-Kludas, Anita Pinar, Weiguang Zeng, Andrea Stutz, Eicke Latz, Lorena E. Brown, Ashley Mansell

**Affiliations:** 1 Department of Microbiology and Immunology, The University of Melbourne, Parkville, Victoria, Australia; 2 Centre for Innate Immunity and Infectious Diseases, Monash Institute of Medical Research, Monash University, Clayton, Victoria, Australia; 3 Institute of Innate Immunity, University Hospitals, University of Bonn, Bonn, Germany; 4 Department of Infectious Diseases and Immunology, University of Massachusetts Medical School, Worcester, Massachusetts, United States of America; Harvard Medical School, United States of America

## Abstract

The ability for a host to recognize infection is critical for virus clearance and often begins with induction of inflammation. The PB1-F2 of pathogenic influenza A viruses (IAV) contributes to the pathophysiology of infection, although the mechanism for this is unclear. The NLRP3-inflammasome has been implicated in IAV pathogenesis, but whether IAV virulence proteins can be activators of the complex is unknown. We investigated whether PB1-F2-mediated activation of the NLRP3-inflammasome is a mechanism contributing to overt inflammatory responses to IAV infection. We show PB1-F2 induces secretion of pyrogenic cytokine IL-1β by activating the NLRP3-inflammasome, contributing to inflammation triggered by pathogenic IAV. Compared to infection with wild-type virus, mice infected with reverse engineered PB1-F2-deficient IAV resulted in decreased IL-1β secretion and cellular recruitment to the airways. Moreover, mice exposed to PB1-F2 peptide derived from pathogenic IAV had enhanced IL-1β secretion compared to mice exposed to peptide derived from seasonal IAV. Implicating the NLRP3-inflammasome complex specifically, we show PB1-F2 derived from pathogenic IAV induced IL-1β secretion was Caspase-1-dependent in human PBMCs and NLRP3-dependent in mice. Importantly, we demonstrate PB1-F2 is incorporated into the phagolysosomal compartment, and upon acidification, induces ASC speck formation. We also show that high molecular weight aggregated PB1-F2, rather than soluble PB1-F2, induces IL-1β secretion. Furthermore, NLRP3-deficient mice exposed to PB1-F2 peptide or infected with PB1-F2 expressing IAV were unable to efficiently induce the robust inflammatory response as observed in wild-type mice. In addition to viral pore forming toxins, ion channel proteins and RNA, we demonstrate inducers of NLRP3-inflammasome activation may include disordered viral proteins, as exemplified by PB1-F2, acting as host pathogen ‘danger’ signals. Elucidating immunostimulatory PB1-F2 mediation of NLRP3-inflammasome activation is a major step forward in our understanding of the aetiology of disease attributable to exuberant inflammatory responses to IAV infection.

## Author Summary

Influenza virus is a highly contagious respiratory pathogen that can cause pandemics, resulting in the deaths of millions worldwide. Previously we demonstrated that PB1-F2 protein produced by pathogenic influenza induces overwhelming inflammatory responses to infection, which enhances disease. The way in which PB1-F2 causes this overt inflammation is unclear. Recently, influenza virus was shown to be involved in activating the inflammasome, which plays a pivotal role during inflammatory responses to infection. However, whether virulence factors such as PB1-F2 produced by the virus can play a role in activation of the inflammasome is unknown. Here, we investigated whether PB1-F2 could have a role in activation of the inflammasome. Using detection of the inflammatory cytokine IL-1β as a marker for inflammasome complex activation, we definitively show PB1-F2 from a pathogenic strain rapidly induces activation of the inflammasome in humans and mice. Using macrophages from mice lacking components of the inflammasome complex, induction of inflammation was shown to be Caspase-1 and NLRP3-dependent. Inflammation induced by PB1-F2 was abrogated in NLRP3-deficient mice. To our knowledge, this is the first description of the mechanism of PB1-F2-mediated inflammasome complex activation. Our work provides further understanding of the contribution of PB1-F2 to enhancing inflammation during influenza infections.

## Introduction

Influenza A virus (IAV) is a major cause of respiratory tract infections and may result in severe immunopathology characterised by high oxidative stress, hypercytokinemia and acute respiratory distress syndrome [1]. Understanding the molecular basis for disease severity for emerging influenza viruses is essential to developing better treatments and improving clinical outcomes in acute infections. Innate recognition of IAV through pattern recognition receptors (PRRs) plays a central role in generating inflammatory responses during infection and the recruitment of infiltrating leukocytes to the lung. Recent studies have implicated several PRRs in recognizing and inducing inflammation in response to IAV challenge [2].

Inflammasomes are cytoplasmic multiprotein complexes that mediate proteolytic processing of interleukin (IL)-1 family members to their mature active form [3]. NOD-like receptors (NLRs) are involved in activating the inflammasome and play a pivotal role during host responses to IAV infection [2]. IAV infection activates the NLRP3 inflammasome complex [4] which consists of the apoptotic speck-like protein containing a caspase activation and recruitment domain (ASC), NLRP3 and caspase-1. Inflammasome-induced cytokine release requires two signals: (1) priming of cells by activating the prototypic inflammatory transcription factor NF-κB that mediates synthesis of pro-IL-1β and upregulation of components of the NLRP3 inflammasome; and (2) triggering of inflammasome formation, which results in IL-1β maturation and secretion. Formation of the oligomeric inflammasome can be triggered by a variety of stimuli that cause membrane perturbations and cellular dysfunction, such as pore forming toxins, ATP, protein amyloid aggregates and crystalline material [5], [6], [7].

Activating the inflammasome complex is important in combating infection as mice deficient in ASC, caspase-1 or IL-1R, display delayed clearance of IAV infection [2], [8]. In addition to influenza virus RNA [9], the IAV M2 ion channel protein has been implicated as an activator of the inflammasome complex, causing the release of mature IL-1β during infection [10], [11]. However, it is unknown whether IAV virulence proteins can contribute to inflammasome activation, which may enhance disease pathology.

The non-structural IAV PB1-F2 protein is associated with virulence [12], [13]. PB1-F2 proteins derived from 20^th^ century pandemic and highly pathogenic IAV strains, but not mildly pathogenic seasonal IAVs, trigger exuberant inflammatory responses in the lungs of infected mice [14]. This response is characterized by enhanced bronchiolar cellular infiltrate, comprising mainly macrophages and neutrophils early during infection [15]. Moreover, this overt inflammatory response has been linked to predisposing infected hosts to bacterial pneumonia [12]. Recently, the secondary structure of PB1-F2 adopted under membranous solution conditions has been correlated with the pathogenicity of the viruses from which the proteins are derived [16]. Amyloid fibers play a role in multiple diseases and have been demonstrated to activate the NLRP3 inflammasome complex [17]. PB1-F2 protein conformations can include β-sheet aggregates, α-helical structures and random coils, which depend upon the environmental conditions and virus isolate [16], [18]. The C-terminal region of PB1-F2 proteins of pathogenic IAV strains form aggregates, similar to amyloid fibers and are thought to contribute to recognition of a structural signature by host pattern recognition receptors. Therefore, we hypothesised that PB1-F2 protein derived from pathogenic IAV contributes to exuberant inflammatory host responses by activating the inflammasome complex. Using reverse-engineered IAV that express PB1-F2 derived from the A/Puerto Rico/8/34 (PR8) isolate, which is highly pathogenic for mice, and an otherwise isogenic virus genetically modified for ablated PB1-F2 production, our studies show that PB1-F2-deficient IAV results in decreased IL-1β secretion and inflammatory cell recruitment in infected mice. We demonstrate that C-terminal PR8 PB1-F2 peptide alone, which was previously shown to potently increase inflammation in the mouse model [14], induces IL-1β secretion, suggesting activation of the inflammasome complex. Additionally, PR8 PB1-F2 peptide induces robust IL-1β secretion in both human PBMCs and murine macrophages. In agreement with Solbak *et al* [16], C-terminal peptide derived from the seasonal, less virulent A/Wuhan/359/1995 (Wuhan) isolate was unable to form aggregates and did not induce inflammasome activation or enhance immunopathology in the lungs. Importantly, NLRP3-deficient mice display significantly lower IL-1β and TNFα production and decreased leukocyte and neutrophil cell infiltration into the airspaces following PR8 PB1-F2 peptide administration *in vivo*. This is the first description of a mechanism by which PB1-F2 can activate host inflammation and cellular responses to infection. Our findings are important in understanding the aetiology of disease severity caused by influenza virus.

## Results

### Increased cellularity and IL-1β secretion in the lungs is caused by PB1-F2

**Figure 1:**
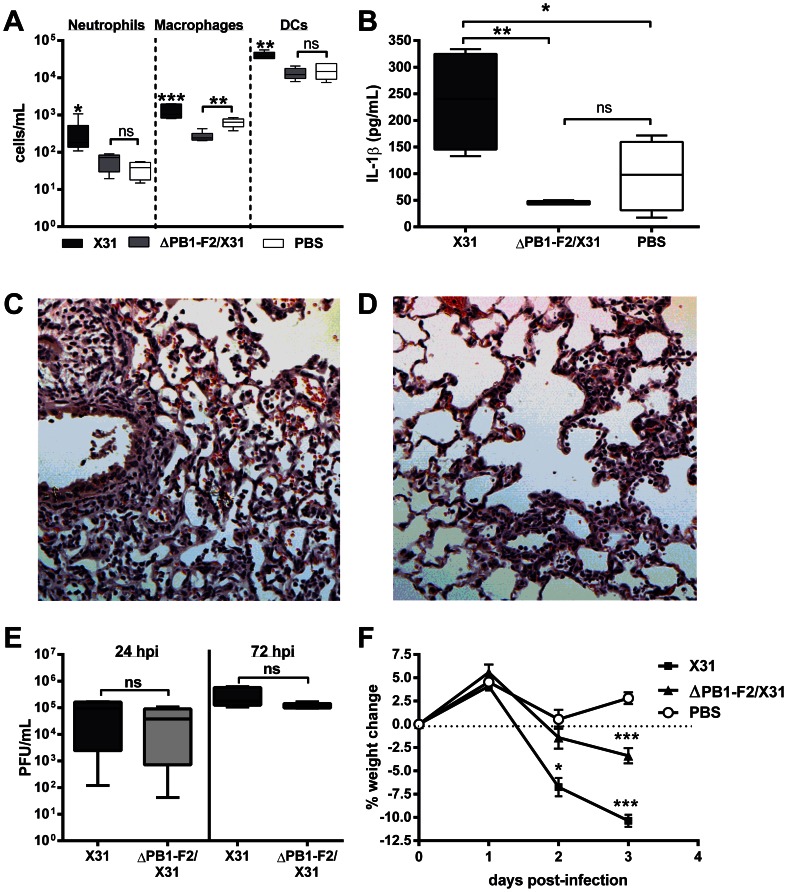
PR8 PB1-F2 increases cellularity and IL-1β secretion during infection in the lungs. Mice (n = 5, 6–8 weeks, C57BL/6) were intranasally infected with 100 PFU X31 or ΔPB1-F2/X31 virus or PBS and BAL-F was collected at 24 hpi. Figure shows **A**) cellular content of the BAL-F examined by flow cytometry for neutrophils, macrophages and dendritic cells (DCs); **B**) IL-1β secreted into the BAL-F determined by ELISA. See also Figure S1. Results show the mean ± SEM and are representative of two independent experiments. *** p<0.001, ** p<0.01, * p<0.05 and ns = p>0.05, ANOVA Tukey post hoc, compared to all other groups, or as indicated; **C**) A representative hematoxylin and eosin stained section of lung tissue obtained from a mouse infected with a sub-lethal dose of X31 at 72 hpi. The section shows perivascular infiltration of lymphocytes, macrophages and neutrophils into interstitial regions, areas of focal necrosis of terminal airways and prominent cellular debris associated with acute hemorrhage into alveoli. The number of foci of inflammatory cells seen in both the interstitium and alveoli was noticeably greater in these mice than in mice infected with **D**) ΔPB1-F2/X31 at 72 hpi. **E**) Lung viral titers (PFU/mL) of mice infected with either X31 or ΔPB1-F2/X31 were determined by plaque assay at 24 h and 72 h post-infection (ns: p>0.05 Student's unpaired T-test at each time point). **F**) The percentage change from the starting weight of mice infected with X31 or ΔPB1-F2/X31 (**p<0.01, ***p<0.001 compared to all other groups on that day, ANOVA Tukey post-hoc).

The induction of inflammation by PB1-F2 protein expressed by reverse engineered A/Puerto Rico/8/34 (PR8) IAV of the H1N1 subtype is well characterized and contributes to the pathophysiology of disease [12], [13], [14]. Here we used reverse engineered X31 virus, which is less lethal than PR8 virus to the C57BL/6 mice used in this study and also shows a higher rate of infection of macrophages. The X31 virus contains H3N2 surface antigens and all other proteins, including PB1-F2, are from PR8. Infection of mice with X31 virus shows similar levels of inflammation as does infection with PR8 virus as determined by equivalent levels of cellular infiltrate in the airways at 24 and 72 hours post infection (hpi) (data not shown). Reverse engineered X31 IAV is a well-established model to characterise influenza immunity [19]. An otherwise isogenic virus with abrogated PB1-F2 production (ΔPB1-F2/X31) was also created to investigate the contribution of PR8 PB1-F2 expression by the X31 virus to recruitment of effector cells to the lungs of C57BL/6 mice following viral infection (Figure 1). Similar to our previously published PR8 data [20], [21], mice infected with ΔPB1-F2/X31 demonstrated a significant decrease in neutrophils, macrophages and dendritic cells (DCs) in bronchoalveolar lavage fluid (BAL-F) 24 h post-infection (hpi) (Figure 1A) compared to mice infected with X31. Mice infected with ΔPB1-F2/X31 continued to have significantly less cellular infiltrate in their BAL-F compared to mice infected with X31 even at 72 hpi (Figure S1A). The decreased cellular infiltrate correlated with decreased IL-1β secretion within BAL fluid (BAL-F) at 24 (Figure 1B) and 72 hpi (Figure S1B). Interestingly, induction of IL-1β mRNA levels were comparable between X31-, ΔPB1/X31- and PBS-treated mice lungs 48 hpi (Figure S1C), suggesting the decrease in IL-1β secretion was not due to differences in viral-induced IL-1β expression but maturation. Histological analysis of infected lung tissue also showed decreased inflammation at 72 hpi in the absence of PB1-F2 expression compared to X31 infected mice (Figure 1C and D respectively). Importantly, X31 and ΔPB1-F2/X31 viruses replicated to the same level at 24 h and 72 h post-infection (Figure 1E). Indicating the enhanced inflammation may be a correlate of disease, mice infected with X31 virus typically lost more weight within 72 hpi than mice infected with ΔPB1-F2/X31 (Figure 1F).

**Figure 2:**
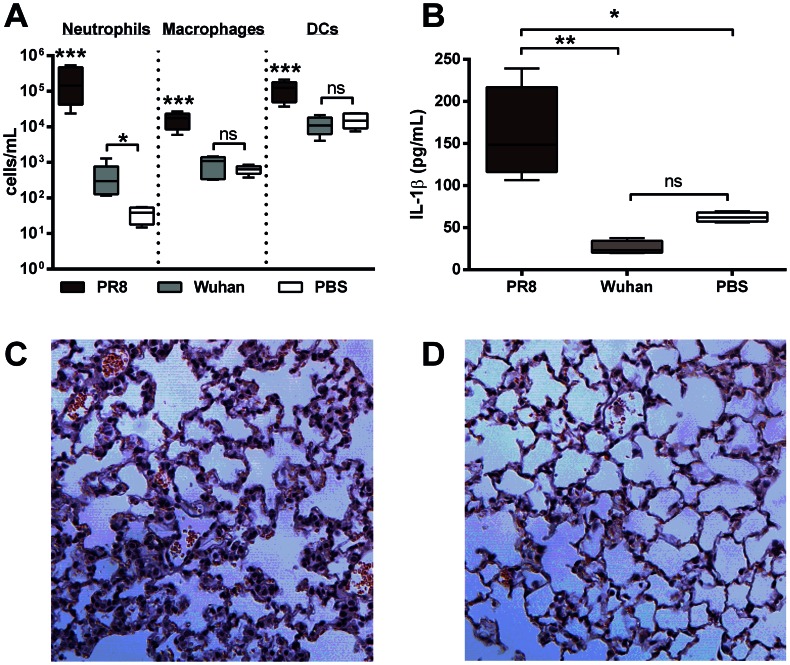
PB1-F2 peptide derived from pathogenic IAV increases cellularity and IL-1β secretion in the lungs. Mice (n = 5, 6–8 weeks, C57BL/6) were intranasally exposed to 100 µg PR8 or Wuhan peptide or PBS and BAL-F was collected at 24 hpi. Figure shows **A**) cellular content of the BAL-F examined by flow cytometry for neutrophils, macrophages and dendritic cells (DCs); **B**) IL-1β secreted into the BAL-F determined by ELISA. See also Figure S1. Results show the mean ± SEM and are representative of two independent experiments. *** p<0.001, ** p<0.01, * p<0.05 and ns = p>0.05, ANOVA Tukey post hoc, compared to all other groups, or as indicated; **C**) A representative hematoxylin and eosin stained section of lung tissue obtained from a mouse 24 h after exposure to PR8 peptide. Analysis revealed similar cellular responses compared to mice infected with X31 at the same time point (not shown). The section shows diffuse infiltration of inflammatory cells into the interstitium and alveoli compared to **D**) minimal rare, perivascular infiltration of lymphocytes in mice exposed to peptide derived from the H3N2 seasonal strain A/Wuhan/359/1995.

To examine the inflammatory response to PB1-F2 without the influence of other IAV viral proteins such as the M2 protein, mice were intranasally exposed to a peptide corresponding to the C-terminal amino acid sequence PB1-F2 (amino acids 60–87 inclusive) of either PR8 or the seasonal non-pathogenic H3N2 strain A/Wuhan/359/1995 and were euthanized 24 or 72 h after inoculation. Consistent with our earlier study [14], administration of the C-terminal PR8 PB1-F2 peptide induced an influx of neutrophils, macrophages and DCs (Figure 2A) into the BAL-F of C57BL/6 mice, at both 100 µg (Figure 2A) and 5 µg (Figure S1D) doses of peptide. Importantly, the cellular infiltrate in mice given 100 µg of the PR8 peptide was significantly greater than in mice given the corresponding Wuhan peptide. The greater cellular infiltrates in PR8 peptide-exposed mice were accompanied by markedly higher levels of IL-1β in the BAL-F 24 h post-exposure to 100 µg (Figure 2B) and 5 µg (Figure S1E) peptide. As expected, mice exposed to PR8 peptide revealed more severe pathophysiology in the lung tissue, compared to those exposed to Wuhan peptide as early as 24 h post-exposure (Figure 2C and D respectively).

### Aggregated PR8 PB1-F2 peptide potently induces IL-1β production in macrophages

**Figure 3:**
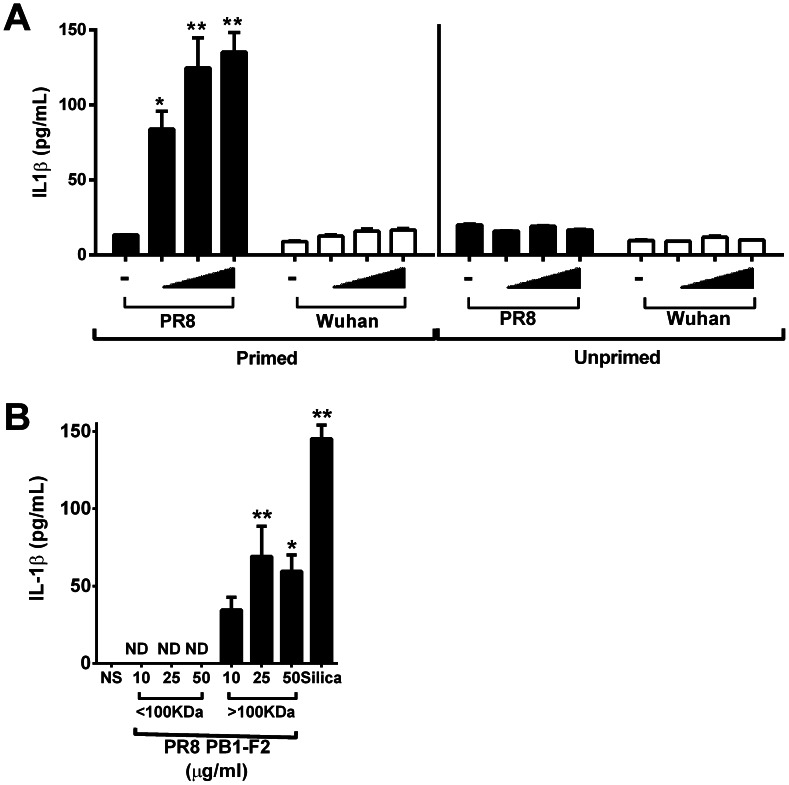
PR8 PB1-F2 aggregates activate the NLRP3 inflammasome. **A**) Wild-type immortalized bone marrow derived macrophages (BMMs) were either primed or not with 100 ng/mL of LPS for 3 h prior to stimulation with a range of doses (10–50 µg/mL) of PR8 PB1-F2 or Wuhan PB1-F2 peptide as indicated for a further 6 h. Results show LPS priming of BMMs was required to induce IL-1β secretion in wild-type cells by PR8 PB1-F2 peptide. * p<0.05 and ** p<0.01 compared to negative (non-stimulated, -) control, ns = not significant, ANOVA Dunnett's Multiple Comparison test. **B**) Immortalized BMMs were primed with LPS and treated with indicated doses of molecular weight fractionated PR8 PB1-F2 for 6 h. Cellular supernatants were collected and analyzed for IL-1β secretion by ELISA according to manufacturer's instructions. Results are representative of three independent experiments and are represented as mean ± SEM. ND = not detectable. * p<0.05 and ** p<0.01 compared to NS (non-stimulated) control, ANOVA Dunnett's Multiple Comparison test.

To examine the inflammatory response to PB1-F2 further, we challenged the wild-type bone marrow derived macrophages (BMMs) with PR8 or Wuhan C-terminally derived PB1-F2 peptides in a dose-dependent manner and analyzed the induction of IL-1β secretion by LPS-primed and unprimed cells (Figure 3A). In a manner consistent with other crystalline or protein aggregates [22] we found that LPS priming of BMMs was required to induce IL-1β secretion in wild-type cells by PR8 PB1-F2 peptide (p<0.001 primed vs unprimed, ANOVA Tukey post-hoc for all doses of peptide, Figure 3A). The Wuhan PB1-F2 peptide did not induce IL-1β secretion either in the presence or absence of LPS priming (p>0.05 primed vs unprimed, ANOVA Tukey post-hoc, Figure 3A). To elucidate the activating form of PB1-F2, we next prepared samples by size fractionation, generating aggregated samples (>100-kDa) or oligomeric samples (<100-kDa). We found that only aggregated PB1-F2 (>100-kDa) was able to induce IL-1β secretion in macrophages (Figure 3B), suggesting the high molecular weight aggregated PB1-F2 specifically induces IL-1β secretion.

### PR8 PB1-F2 induces IL-1β secretion via caspase-1 and the NLRP3 inflammasome

**Figure 4:**
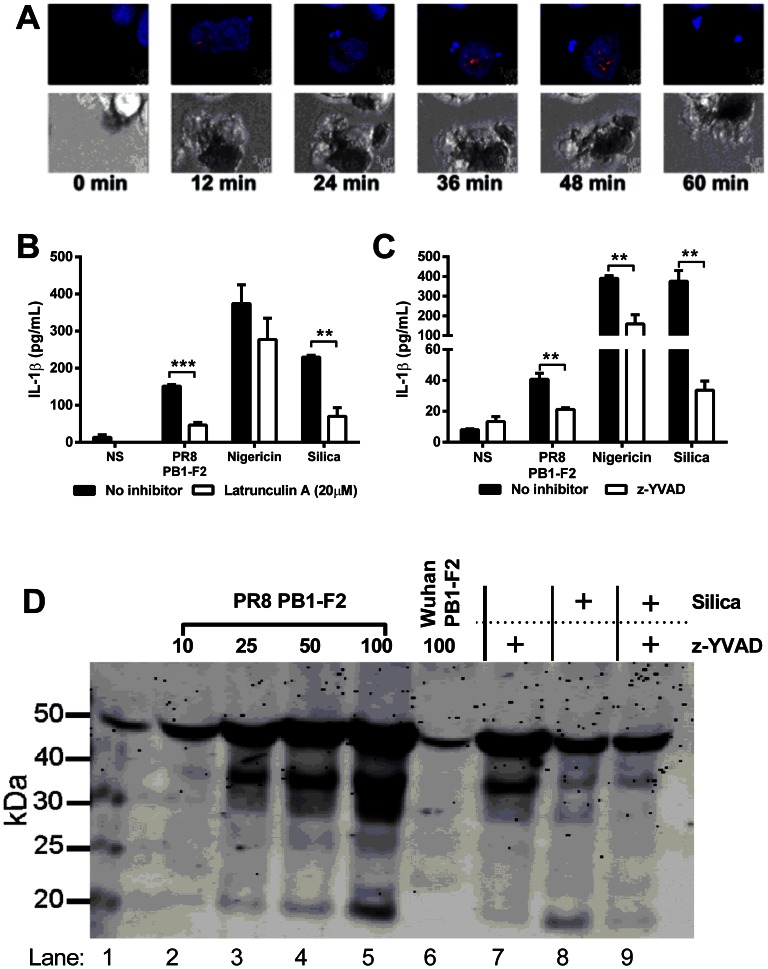
PR8 PB1-F2-mediated inflammation is NLRP3-, Caspase 1- and phagocytosis- dependent and induces ASC speck formation in macrophages. **A**) Immortalized NLRP3-deficient macrophages stably reconstituted with ASC-cerulean (blue) and NLRP3 were seeded at 10^5^/mL in 35 mm glass bottom dishes 24 h prior to stimulation with pHrodo-labeled (red) PB1-F2 peptide. Time-lapse imaging was performed for 3.5 h. Representative images shown are flattened maximum intensity projections of 3D deconvolved z-stacks using Imaris. Scale bar is 3 µm. See Video S1 and S2. Immortalized BMMs were primed with LPS, then **B**) pre-treated with 20 µM Latrunculin A (Lat A) for 45 min to inhibit phagocytosis, prior to stimulation with PR8 PB1-F2, nigericin (10 µM), silica (125 µg/mL), or unstimulated (NS) for 6 h; **C**) pre-treated with 10 µM caspase-1 inhibitor z-YVAD for 45 min, prior to stimulation with PR8 PB1-F2 (50 µg/mL), nigericin (10 µM), silica (125 µg/mL), or unstimulated (NS) for 6 h. Cellular supernatants were collected and analyzed for IL-1β secretion by ELISA according to manufacturer's instructions. Results are representative of three independent experiments and are represented as mean ± SEM. ** p<0.01 and *** p<0.001, Unpaired t-test. **D**) Immortalized wild-type macrophages were seeded at 2×10^6^ cells in 6 well plates, 24 h prior to 3 h priming with LPS (200 ng/ml) in serum free media. Cells were pretreated (where indicated) with the caspase-1 inhibitor z-YVAD (20 µM) for 40 mins and then exposed to either PR8 or Wuhan PB1-F2 (50 µg/mL), or silica (125 µg/ml) for 6 h. Proteins were concentrated from cultured supernatants and separated by 4–12% SDS-PAGE, transferred to nitrocellulose and immunoblotted for caspase-1 cleavage. Result represents one of three independent experiments.

To determine if phagocytosis of the PB1-F2 peptide is required for activation of the inflammasome in living cells, the PR8 PB1-F2 peptide was labeled with the pH-sensitive dye pHrodo, which dramatically increases in fluorescence intensity in the acidic environment of the phagolysosomal compartment [23]. NLRP3-deficient macrophages, stably reconstituted with cerulean-tagged ASC and NLRP3-Flag, which obviates the need to prime the macrophages, were treated with the labeled peptide and ASC speck formation visualized as a well-characterized marker of inflammasome formation and activation of caspase-1 [24]. As observed in Figure 4A and live cell imaging (Video S1), pHrodo-labeled PB1-F2 peptide is rapidly phagocytosed into the lysosomal pathway as visualized by increased red fluorescence of PB1-F2 within cellular vesicles. This is followed by ASC speck formation indicative of activation of the inflammasome complex. These events occur within the majority of cells treated with labeled PR8 PB1-F2 peptide (Video S2). Consistent with earlier data, experiments with pHrodo-labeled PB1-F2, separated into molecular weight fractions and added to cells, demonstrated that only the higher molecular weight (>100 kDa) fraction is phagocytosed as evidenced by increased red fluorescence and ASC speck formation, whereas the lower molecular weight fraction (<100 kDa) had no effect on the cells (Figure S2). Importantly, inhibition of phagocytosis with the actin polymerization inhibitor Latrunculin A inhibited pHrodo-PB1-F2 uptake in ASC-cerulean cells (Video S3). Furthermore, Latrunculin A inhibited PB1-F2-induced IL-1β secretion in macrophages (Figure 4B), whereas nigericin, a potassium ionophore known to activate the inflammasome but not requiring actin polymerization [25], was unaffected. Silica however, which does require actin polymerization for inflammasome activation and IL-1β maturation [6] was sensitive to Latrunculin A treatment. Together these results clearly demonstrate that phagocytosis is required for PB1-F2 peptide-induced inflammasome formation.

PR8 PB1-F2 peptide induced robust IL-1β secretion, comparable to other activators of the inflammasome including nigericin and silica, which was inhibited when cells were treated with caspase-1 inhibitor z-YVAD (Figure 4C). Immunoblot analysis of caspase-1 demonstrated that PR8 PB1-F2 induced the proteolytic cleavage of the active caspase-1 protein in a dose dependent manner comparable to that observed for silica (Figure 4D). Importantly Wuhan PB1-F2 did not induce caspase-1 cleavage (see lane 6). PR8 PB1-F2- and silica-mediated cleavage of caspase-1 were both inhibited by z-YVAD (compare lane 7 to lane 5 and lanes 6 and 7 respectively).

**Figure 5:**
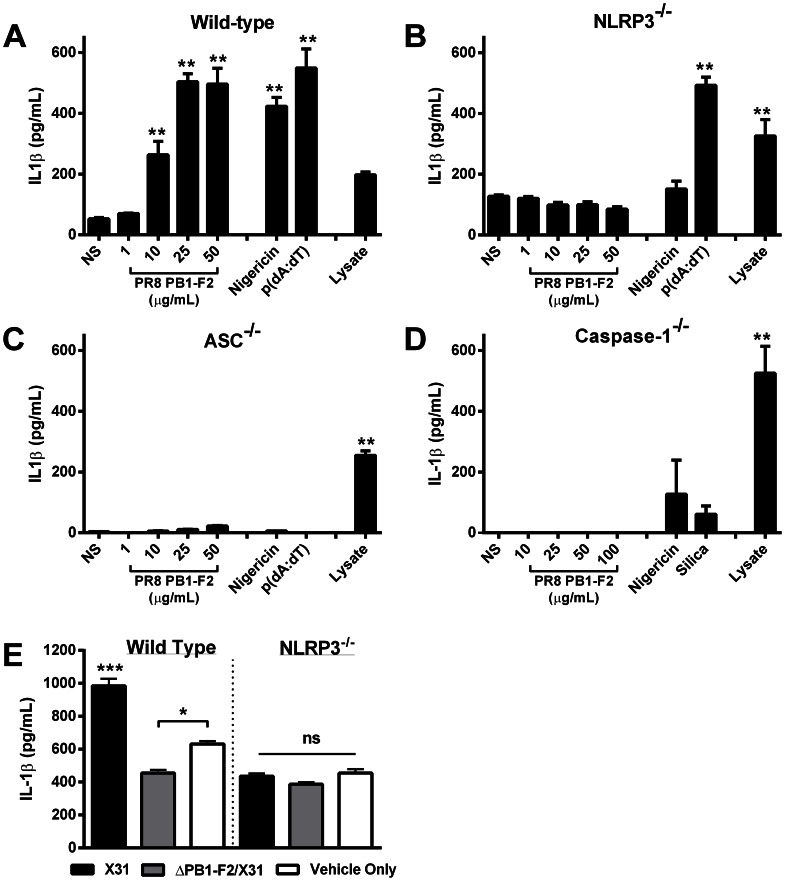
Murine cells unable to express NLRP3-inflammasome complex activators are not stimulated by PR8 PB1-F2 peptide. Bone marrow derived macrophages derived from **A**) wild-type, **B**) Caspase-1, **C**) ASC, and **D**) NLRP3-deficient mice were primed with LPS (100 ng/mL) for 3 h and then stimulated with PR8 PB1-F2 peptide, nigericin (10 µM), silica (125 µg/mL), or transfected with poly (dA∶dT) (250 ng/mL), or left unstimulated (NS) for a further 6 h. Priming of cells was confirmed by disrupting cellular membranes of unstimulated cells by repeated freezing and analysis of cellular lysate IL-1β concentrations. Cellular supernatants were collected and analyzed for IL-1β secretion by ELISA according to manufacturer's instructions. Results are representative of three independent experiments and are represented as mean ± SEM. **p<0.01 compared to NS, ANOVA Dunnett's Multiple Comparison Test. **E**) Wild type and NLRP3-deficient macrophages were primed with 100 ng/mL of LPS for 3 h prior to infection with 10MOI X31, ΔPB1-F2/X31 or infection media (vehicle only). Cellular supernatants were collected and analyzed for IL-1β secretion by ELISA. * p<0.05, *** p<0.001 ANOVA, Tukey post-hoc.

To demonstrate PR8 PB1-F2 peptide activation of the NLRP3 inflammasome complex, we used immortalized BMMs derived from mice deficient in caspase-1, ASC or NLRP3 [6], [26] and their immortalized wild-type counterparts, and repeated our PR8 peptide exposure experiments. As expected, PR8-PB1-F2 peptide induced IL-1β secretion in a dose dependent manner, comparable to other known NLRP3 and AIM2 activators of the inflammasome, including nigericin and poly(dA∶dT) respectively (Figure 5A). Exposure of wild-type macrophages to 25 µg or 50 µg PR8 PB1-F2 peptide induced an equivalent amount of IL-1β secretion as nigericin and poly(dA∶dT) (p>0.05 ANOVA Tukey post-hoc). Caspase-1 dependency was supported by the abrogation of IL-1β secretion by PR8 PB1-F2 in caspase-1-deficient macrophages (Figure 5D). Macrophages lacking either ASC (Figure 5C) or NLRP3 (Figure 5B) also failed to secrete IL-1β in response to PR8 PB1-F2 peptide, suggesting a requirement of both these inflammasome components in processing IL-1β. Validating this data and consistent with earlier reports [27], cells exposed to nigericin, a microbial toxin that acts as a potassium ionophore, revealed an ASC- and NLRP3-dependent response (Figure 5C and 5D), whereas the response to transfected poly(dA∶dT) was only ASC-dependent (Figure 5C), consistent with its requirement as an AIM2-dependent inflammasome activator. Finally, we confirmed the requirement of NLRP3 in PB1-F2 peptide induced IL-1β secretion in peritoneal macrophages obtained from wild-type and NLRP3-deficient mice (Figure S3). Exposure of wild-type and NLRP3−/− cells to PB1-F2 peptide induced a dose dependent cell death (10–35%) that was comparable between both genotypes (data not shown).

To further examine whether PR8 PB1-F2 is able to activate the NLRP3 inflammasome, we examined whether production of PB1-F2 could induce IL-1β secretion in IAV infected wild-type and NLRP3-deficient macrophages. The data show that primed wild-type cells infected with X31 virus but not with ΔPB1-F2/X31 yielded significant IL-1β production above cells treated with medium alone, again highlighting the PB1-F2 dependence of the phenomenon (Figure 5E). In contrast, infection of primed NLRP3^−/−^ cells with X31 showed no significant IL-1β production compared to the medium control, showing the NLP3-dependence of influenza virus-induced IL-1β production.

The dependency of both priming and expression of NLRP3 in order for X31 to cause increased IL-1β indicates PB1-F2 protein expression is a potent activator of signal 2 of the NLRP3-inflammasome complex. Collectively, these results demonstrate that PR8 PB1-F2 induces proinflammatory IL-1β secretion via an inflammasome consisting of ASC, NLRP3 and caspase-1.

### NLRP3 deficiency ablates PB1-F2 induced inflammation

**Figure 6:**
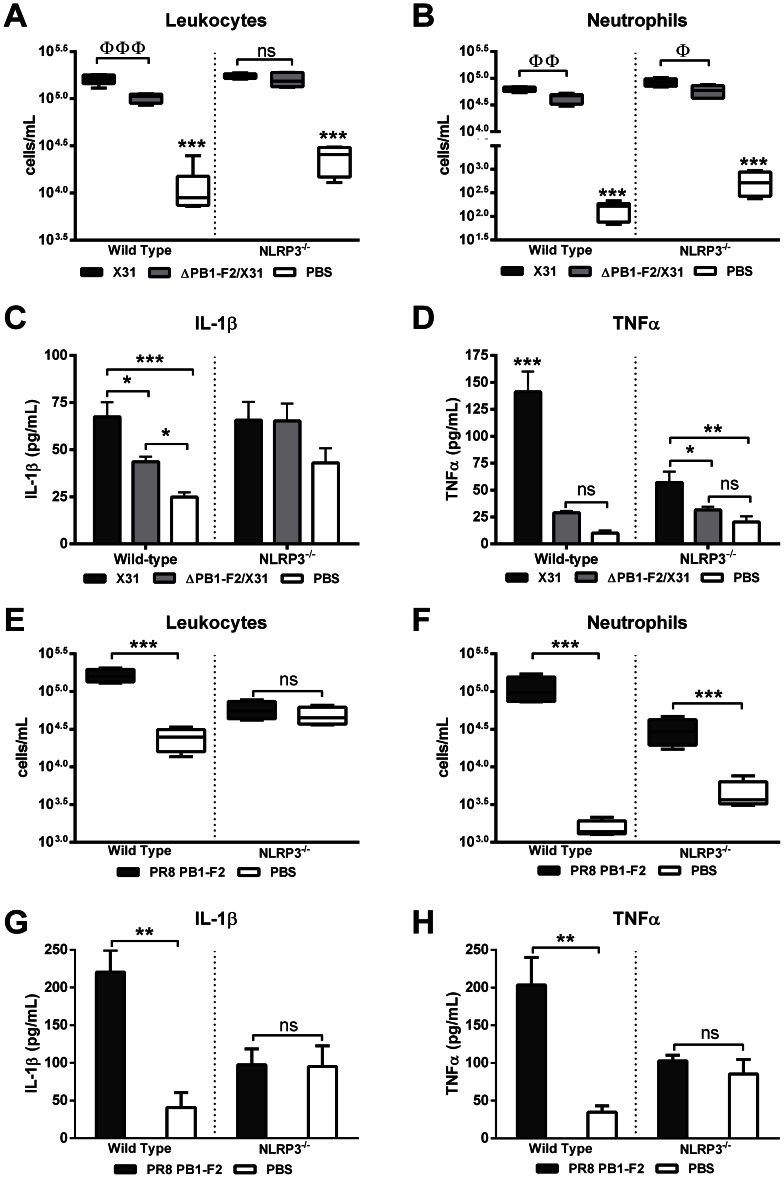
Induction of inflammasome activation by PR8 PB1-F2 peptide occurs rapidly *in vivo*. Wild-type and NLRP3-deficient (*NLRP3^−/−^*) mice (n = 4–5 per group) were intranasally infected with 100 PFU X31 or ΔPB1-F2/X31 virus, or treated with PBS or 100 µg PR8 PB1-F2 peptide. BAL-F was assessed 2 d later for virus infected and 5 h later for peptide challenged mice, by flow cytometry for detection of **A**) and **E**) leukocytes (CD45^+^) and **B**) and **F**) neutrophils; and via ELISA for **C**) and **G**) IL-1β and **D**) and **H**) TNFα levels. These data are representative of two independent experiments. Error bars represent mean ± SEM. ΦΦΦ p = 0.0004, ΦΦ p = 0.0069, Φ p = 0.0243 Student's unpaired T-test; ns: p>0.05, *p<0.05, **p<0.01 and ***p<0.001 one-way ANOVA Tukey post-hoc.

To investigate PB1-F2 activation of the NLRP3 inflammasome complex *in vivo*, we challenged NLRP3-deficient mice with X31 or ΔPB1-F2/X31 viruses, or PR8 PB1-F2 peptide or PBS (as a negative control) and compared cellular secreted IL-1 β and the prototypic NF-κB-dependent inflammatory cytokine TNFα responses to those of wild-type mice. Importantly, groups of infected mice had lung viral titres within the range of 10^5.7^±10^0.1^ PFU/mL (p>0.05 student's unpaired T-test) at 2 d post-infection with either X31 or ΔPB1-F2/X31 virus. There was negligible weight loss in the infected WT and NLRP3−/− mice at 2 d post-infection (data not shown). As expected, wild-type mice infected with virus expressing PB1-F2 (X31) or exposed to PR8 PB1-F2 peptide induced an enhanced presence of leukocytes and neutrophils in the BAL-F compared to wild-type mice infected with ΔPB1-F2/X31 (Figure 6A and B), or PBS challenge (Figure 6E and F) respectively. Consistent with our earlier data, wild type mice infected with X31 virus display an approximately 3-fold increase in IL-1β secretion while ΔPB1-F2/X31 viral challenge induces only a 2-fold increase as compared to PBS challenged mice.

Confirming PB1-F2 activation of the inflammasome complex is NLRP3 dependent, NLRP3-deficient mice infected with X31 virus or ΔPB1-F2/X31 virus display a non-significant 1.5 fold increase in IL-1β secretion (Figure 6C) compared to PBS challenged NLRP3-deficient mice. Moreover, mice infected with X31 virus had elevated levels of TNFα compared to those infected with ΔPB1-F2/X31 (Figure 6D), which is consistent with our IL-1β observations. Linking the C-terminal domain of PB1-F2 directly with NLRP3-dependent inflammasome activation *in vivo*, we noted that wild type mice challenged with PB1-F2 peptide demonstrated significantly more leukocyte and neutrophil recruitment to the lung following peptide challenge (Figure 6E and 6F). NLRP3-deficient mice challenged with PR8 PB1-F2 peptide resulted in unaltered leukocyte recruitment compared to mice challenged with the PBS control (Figure 6E). However, while NLRP3-deficient mice challenged with PR8 PB1-F2 peptide yielded lower levels of neutrophil recruitment than in the wild-type challenged animals (p<0.001, ANOVA Tukey post-hoc), a significant influx of neutrophils was observed compared to mice challenged with the PBS control, indicating neutrophil recruitment can be a result of NLRP3-dependent and –independent means. This data is consistent with previous studies examining lung leukocyte and neutrophil influx following silica challenge demonstrating NLRP3-depdendent decreased neutrophil influx, but no change amongst other cell types [6]. At the same time point, NLRP3-deficient mice also demonstrated significantly decreased IL-1β and TNFα levels in the BAL-F of PR8 PB1-F2 peptide challenged wild-type mice (p<0.05, ANOVA Tukey post-hoc) as compared to PBS-treated mice (Figure 6G and H respectively). Collectively, these results demonstrate that PB1-F2 can induce a rapid cellular and cytokine response in the respiratory tract that is NLRP3-dependent.

### Inflammatory properties of PB1-F2 are relevant in human infection

**Figure 7:**
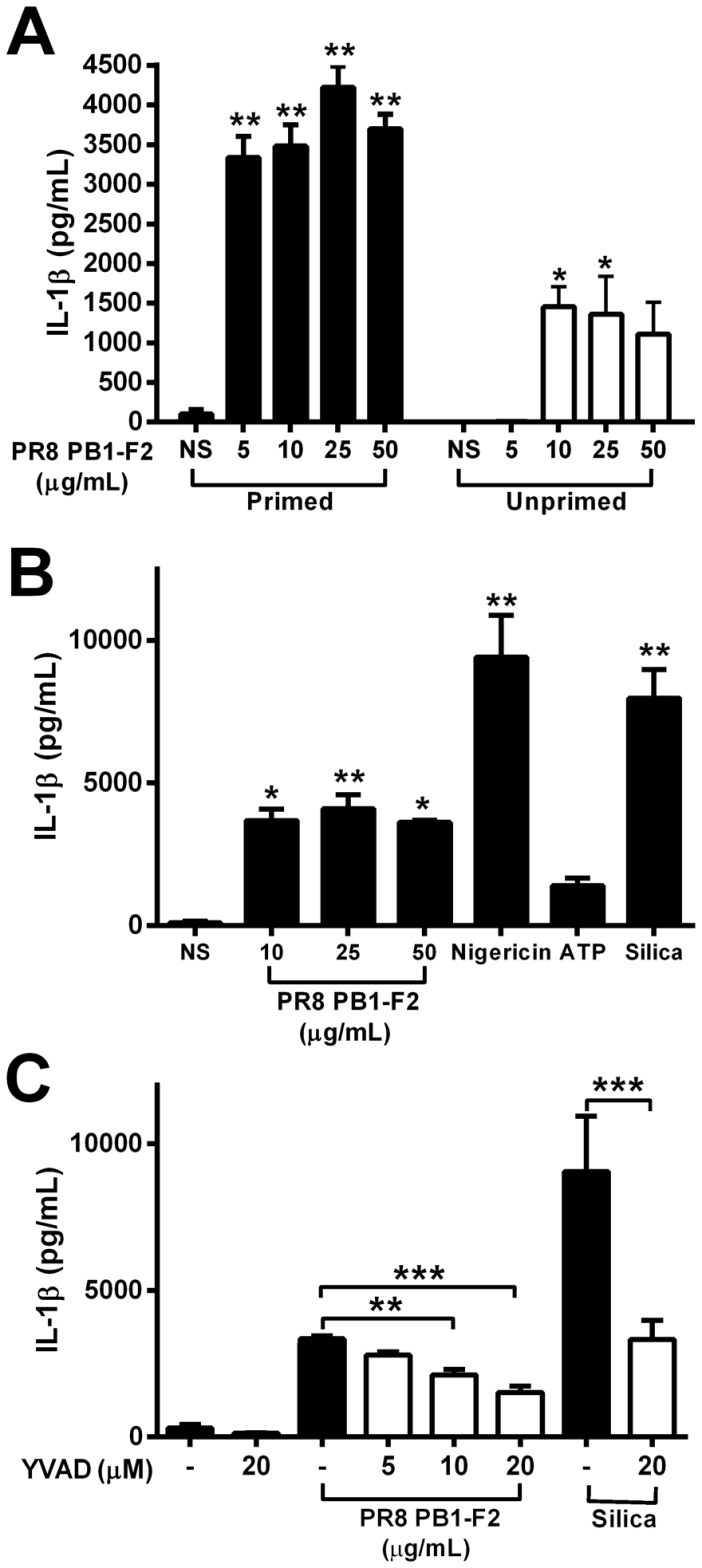
PB1-F2 induction of IL-1β secretion of IL-1β by human PBMCs is caspase-1 dependent. Human PBMCs from three individuals were primed with LPS (50 pg/mL) for 3 h or not, then stimulated with **A**) PR8 PB1-F2 or **B**) PR8 PB1-F2, nigericin (10 µM), silica (125 µg/mL), or ATP (100 mM) for a further 6 h. Cultured supernatants were assayed for IL-1β by ELISA. **C**) LPS-primed hPBMCs were stimulated with PR8 PB1-F2 or silica (125 µg/mL) in the presence or absence of the caspase-1 inhibitor z-YVAD for 6 h. NS = not-stimulated. Results are the mean ± SEM of three independent donors. **p<0.01, ***p<0.001 compared to NS, ANOVA Dunnett's Multiple Comparison Test.

Severe immunopathology resultant of IAV infection is a major contributor to human disease and is characterized by high levels of inflammatory cytokines, chemokines and cellular infiltrates [28]. To evaluate whether expression of PB1-F2 protein by pathogenic IAV may also enhance pathophysiology in humans, we examined cellular responses in human peripheral blood mononuclear cells (hPBMCs) exposed to the PB1-F2 peptide for 6 h. hPBMCs were pre-treated with LPS to induce up-regulation of pro-IL-1β in conjunction with components of the inflammasome, or left unprimed. PR8 PB1-F2 induced IL-1β secretion in unprimed hPBMCs, but primed hPBMCs displayed significantly more IL-1β secretion (Figure 7A). PR8 PB1-F2-induced maturation of IL-1β was as potent as known inflammasome activators such as nigericin, ATP and silica (Figure 7B). Inhibition of caspase-1 enzyme activity with z-YVAD significantly decreased PR8 PB1-F2-induced secretion of IL-1β in a dose-dependent manner, comparable to that observed for z-YVAD inhibition of crystalline silica-induced IL-1β maturation (Figure 7C). These data suggest that PB1-F2 activates IL-1β secretion in a caspase-1-dependent manner in human monocytes.

## Discussion

PB1-F2 derived from pathogenic IAV appears to constitute a novel ‘danger’ signal sensed by the innate immune inflammasome, leading to induction of inflammation. Here, we describe for the first time the mechanism whereby the C-terminal domain of PR8 PB1-F2 protein induces IL-1β production and inflammation via the NLRP3 inflammasome. Our findings clearly implicate PB1-F2 in the recruitment and activation of neutrophils, macrophages and DCs to the airways following viral challenge, a crucial step in the enhancement of pathophysiology during influenza infection [12], [14]. Importantly, we showed that the NLRP3 inflammasome was not only critical to PR8 PB1-F2-induced recruitment of neutrophils and IL-1β production, but was also required for the production of the NF-κB-dependent inflammatory cytokine TNFα. In accordance with our findings, previous reports have reported TNFα release triggered by inflammasome activation is regulated by the IL-1β signaling pathway [7], [29]. Together, our findings indicate that PB1-F2-induced inflammasome activation and subsequent IL-1 signaling is essential in the production of the inflammatory cytokines such as TNFα following IAV infection.

PR8 PB1-F2 has been previously described as a mitochondrially-localized pro-apoptotic protein of monocytes, which antagonises interferon (IFN) signalling by targeting the RIG-I like receptor pathway and co-localization with the mitochondrial antiviral signaling protein (MAVS) [30], [31], [32]. However, these findings do not describe the mechanism by which PB1-F2 appears to induce overt inflammatory responses to IAV infection [12], [14]. We have now described a further role for PB1-F2 protein in triggering host responses that mediate inflammation via induction of the activation of the NLRP3 inflammasome. The differing activities attributed to PB1-F2 may be explained by its structure as a disordered protein, which may switch conformation from random formations to α-helical or β-sheet secondary structures depending upon localized conditions [16], [33]. PR8 PB1-F2 aggregates may favor random β-sheet fibrils that activate the inflammasome, or alternatively, form membrane pores in the mitochondria to disrupt mitochondrial membrane potential and initiate pro-apoptotic pathways [14]. The recent description of PB1-F2 structural signatures between IAV strains may provide insights into the different inflammatory phenotypes observed amongst PB1-F2 expressing strains. The presence or absence of particular ‘signatures’ or amino acids may allow PB1-F2 to form aggregates and thus activate the inflammasome. One explanation for the lack of IL-1β production by cells exposed to Wuhan C-terminal PB1-F2 peptide is that the amino acid sequence does not contain the predicted aggregation motif [16] and thus may not be able to form amyloid fibers under the same conditions that PR8 C-terminal PB1-F2 peptide does. Indeed, the link between PR8 PB1-F2 secondary conformation, mitochondrial membrane potential, mitochondrial dynamics and apoptosis [34] may explain how PR8 PB1-F2 can antagonize viral IFN production and play a role in mitochondrial apoptosis. It has been demonstrated that PB1-F2 inhibits MAVS-mediated IFN production by binding to the MAVS adaptor protein, which may have two effects: (1) a decrease in IFN production and (2) the promotion of VDAC1-mediated cell death [32], [35]. Critically, PB1-F2 has been demonstrated to form fibrillar structures in the cytosol of infected cells [33]. Our real-time demonstration of rapid phagocytosis of PR8 PB1-F2 (Figure 4), incorporation into the lysosomal pathway and subsequent ASC speck formation (Figure S2 and Videos S1-S3), supports a mechanistic role for oligomeric PB1-F2 released from dying infected cells that may be detected by infiltrating leukocytes to induce the inflammasome and drive inflammation. Additionally, only aggregated PB1-F2 (>100-kDa) was able to induce IL-1β secretion in macrophages (Figure 3B), suggesting the high molecular weight species such as fibrillar PB1-F2 could induce IL-1β secretion. Importantly, we demonstrated that in the absence of priming, or NLRP3 inflammasome potentiators including caspase-1, ASC or NLRP3, PR8 PB1-F2 was unable to induce IL-1β secretion. This indicates the disruption of membrane potential by the PR8 PB1-F2 protein that constitutes its pro-apoptotic function is not the causative agent for inflammasome activation. Interestingly, PR8 PB1-F2 was able to induce IL-1β secretion without priming in human peripheral blood mononuclear cells. As PB1-F2 has been associated with mitochondrial disruption and the release of ROS species, PB1-F2 may prime the inflammasome via ROS activation [2] and the induction of IL-1β secretion by aggregated PB1-F2 [33]. The mechanism for the lack of the need for priming hPBMCs to trigger IL-1β secretion upon exposure to PB1-F2 peptide is the focus of currently ongoing studies.

Several viral proteins display a highly disordered structure that allows important functional implementations [36]. The concentration and localization of PB1-F2 during infection of particular cells may direct its conformational structure and functional role. Our data also demonstrates that in addition to viral pore forming toxins, ion channels and RNA activators of the inflammasome, disordered viral proteins may also induce inflammasome activation and act as a pathogen ‘danger’ signal to the host. As disordered viral protein aggregates may be formed during infection with a range of different pathogens, our PR8 PB1-F2 NLRP3 inflammasome activation model may serve as a new tool to investigate interactions between pathogenic infections and host immune responses.

Specific PB1-F2 amino acids that enhance inflammation have been identified at the C-terminus and map to residues 62, 75, 79 and 82, and serine (S) at residue 66 are linked to virulence [37], [38]. The PR8 PB1-F2 peptide used in our studies carried the four “inflammatory” residues, but the Wuhan PB1-F2 peptide did not. Given the marked differences in their abilities to stimulate the inflammasome, PB1-F2's primary or secondary structure may contribute to the inflammatory phenotype. The three pandemic viruses of the 1900s as well as the highly pathogenic avian H5N1 influenza virus all contained the four “inflammatory” residues, but not necessarily 66S. Interestingly, the 2009 H1N1 pandemic virus encodes a truncated PB1-F2 protein and induced milder disease than previous pandemic viruses. This virus, engineered to have the full-length PB1-F2 restored, expresses two of the four inflammatory modulators and was shown to induce only mild illness in swine [39], ferrets and mice [40]. It will therefore be important in future studies to determine if these ‘inflammatory’ residues which are found within the C-terminus of PB1-F2 associated with amyloid formation and aggregation [16], have an impact on inflammasome activation and may further shed light on PB1-F2 structure and function during infection.

Previous studies identifying IAV M2 protein as an inflammatory complex activator [10], [11], may also implicate PB1-F2. Enhanced production of IL-1β was demonstrated in wild-type BMMs infected with either PR8 (H1N1), H3N2 A/Udorn/307/19272, (encoding full-length PB1-F2 protein with the four pro-inflammatory amino acid sites, but not 66S) or A/Guizhou-X (H3N2), a reverse engineered virus containing PB1 from PR8. Reduced IL-1β production was demonstrated for two H1N1 subtype viruses that encoded C-terminally truncated, 56 amino acid length PB1-F2 protein, as well as two H3N2 subtype viruses encoding full length PB1-F2 proteins lacking any of the four inflammatory sites. The M2 deletion virus mutants used in these studies also caused a decrease in IL-1β production, but what effects the abrogation of M2 expression had on normal virion formation and function, as well as expression of the PB1-F2 protein remains unclear. Importantly, our findings show that in the absence of PB1-F2 expression, IAV does not induce robust acute IL-1β production and the initial exuberant cellular response to infection is diminished. As this engineered virus is otherwise isogenic to the unaltered counterpart, it appears that the IAV M2 protein may influence NLRP3 activation in combination with PB1-F2, which may be a virus strain-dependent phenomenon.

The function of the newly discovered N40 protein [41] is unknown. The influence of PB1-F2 on N40 production is very controversial. Of the published studies, Tauber *et al* [42] utilized the reverse engineered WSN strain that cannot produce PB1-F2 and showed that the virus appeared to have an enhanced N40 production. However, Tauba *et al* showed that the WSN virus genetically manipulated to ablate the production of PB1-F2, had slower replication kinetics *in vitro* [42],which is in contrast with Zamarin *et al* in which the modified virus replicated equally well or better than its wild-type WSN counterpart [43]. Additionally, Pena *et al* [44] showed that the presence or absence of PB1-F2 had negligible effects on the level of N40 expression, although this may be an influenza strain dependent phenomenon. The inclusion of our peptide studies is highly important as it negates the influence of any other viral proteins, including N40. Whether the N40 protein influences activation of cellular inflammatory processes is currently under investigation.

Studies have demonstrated an important role for both IL-1β and IL-18 in the pathogenesis of IAV infection. While our data clearly demonstrates a direct role for PB1-F2-induced inflammation and maturation of IL-1β, thus linking the inflammasome and IL-1β with enhanced pathophysiology of pandemic IAV infection, we do not understand how PB1-F2 may regulate IL-18 production. Previously, studies have shown that IL-1β and IL-18 plays a significant role in the innate immune response to IAV challenge, primarily in a protective sense [9], [45], but is less significant for adaptive responses [46]. Consistent with our findings, genetic deletion of components of the inflammasome complex reduce the characteristic cytokine storm associated with relatively pathogenic IAV strains such as PR8, constraining the extent of damage caused by inflammatory cytokines. Given the critical role IL-18 plays in regulating inflammation, induction of the Th1 response characterized by IFNγ production and its role in execution of effective anti-viral immunity [47] future studies should incorporate investigation of the interplay between PB1-F2 and induction of IL-18 immunity,

Importantly, PB1-F2's role in inducing the inflammasome and IL-1β production is relevant to the phenomenon of secondary bacterial infections; the cause of the majority of IAV related mortalities. PB1-F2 predisposes the host to bacterial infection by enhancing the pathophysiology of the inflammatory response [12], [38]. Whether PB1-F2 enhancement of leukocyte influx to the airways and inflammasome activation, leading to production of IL-1β is beneficial or detrimental in the context of pandemic influenza infections requires further investigation. Given PB1-F2 protein inhibits type I IFN production and therefore potentially reduces IFN-mediated inhibition of inflammasome activation [48], the dual roles of PB1-F2 proteins of inflammatory phenotype may contribute to an over exuberant response to infection and predisposition to secondary bacterial infections, resulting in poorer disease outcomes. To our knowledge this study represents the first description of a mechanism of action characterizing PB1-F2 induced inflammation and provides a platform for understanding PB1-F2's role in IAV infections. Our findings additionally suggest that pharmacological intervention aimed at decreasing inflammasome activation and neutrophil recruitment may hold therapeutic promise in cases of primary IAV infections, which may also decrease the incidence of secondary bacterial infections.

## Materials and Methods

### Ethics statement

All experimental procedures were approved by the University of Melbourne Microbiology and Immunology Animal Ethics Committee (AEC) (approval number 911291), or Monash Medical Centre-AEC (approval number MMCA/2012/25) under relevant institutional guidelines, the Prevention of Cruelty to Animals Act 1986 and associated regulations, and the Australian Code of Practice for the Care and Use of Animals for Scientific Purposes.

### Cell lines, viral infections and detection of inflammatory cytokines

Immortalized wild-type C57BL/6 macrophages [40] and primary cells were grown in Dulbecco's Minimal Essential Medium (DMEM, Gibco) supplemented with 10% heat inactivated fetal bovine serum (FBS) and 2 mM glutamine. Cell cultures were maintained at 37°C in a 5% CO_2_ incubator. Cells were washed once with phosphate buffered saline (PBS), infected with the amount of virus indicated by the multiplicity of infection (MOI) in DMEM supplemented with 0.75% bovine serum albumin (BSA, Sigma), 2 mM glutamine, and further incubated as described. For the detection of IL-1β and TNFα, culture supernatants were harvested, debris pelleted at 1,000 g, 5 min, 4°C and 50 µL undiluted sample supernatant used in a mouse IL-1β or TNFα ELISA (Becton Dickinson) according to the manufacturer's specifications. Nigericin and z-YVAD were from Calbiochem, silica (US Silica), Latrunculin A and poly (dA∶dT) were from Sigma-Aldrich.

### Peptide generation

Using the predicted amino acid sequence of the PB1-F2 proteins from PR8 and A/Wuhan/359/1995, peptides from the C-terminal end were synthesized by the Jackson Laboratory (Department of Microbiology & Immunology, University of Melbourne). The peptide region began at amino acid 61 and extended to the termination of the protein (by PR8 sequence) at amino acid 87 as previously described [14]. Immediately prior to use, peptides were resuspended in PBS and used at the concentrations indicated. Importantly, HEK293 cells stably expressing TLR4 and MD2, did not respond to peptide challenge (data not shown), indicating that LPS was not contaminating the peptides, consistent with the need to provide signal 1 to the cells. Fractionation of PB1-F2 was preformed using 100 kDa molecular cut off spin filters from Millipore. PR8 PB1-F2 peptide was labeled with pHrodo (Life Technologies) according to manufacturer's instructions.

### Generation of plasmids and viruses

A set of plasmids were generated on the pHW2000 backbone as described [49], [50], encoding for the PB1, PB2, NP, PA, M and NS gene segments of A/Puerto Rico/8/1934 (PR8) and the HA and NA gene segments of A/Aichi/2/1968. To create the ΔPB1-F2 virus, the open reading frame for PB1-F2 was disrupted by altering the start codon (T120C mutation by PB1 numbering), so translation will not initiate and inserting a stop codon after 11 and 56 residues (C153G and G291A respectively), to ensure complete inability for production of PB1-F2 protein. In no case did the mutations in the PB1-F2 open reading frame cause non-synonymous mutations in the PB1 open reading frame. The N40 start codon [41] was intact in all PB1 plasmids. The wild-type PR8 PB1 plasmid and the ΔPB1-F2/PR8 PB1 plasmid were then incorporated by reverse genetics into virus containing the HA and NA of A/Aichi and PB2, NP, PA, M and NS of PR8, as described [50]. Resulting X31 and ΔPB1-F2/X31 viruses were rescued by one passage in Madin Darby Canine Kidney (MDCK) cells, then propagated a single time in egg stocks. All viruses were fully sequenced to ensure no inadvertent mutations occurred during virus rescue and propagation, then characterized in tissue culture and eggs as previously described [50]. Expression, or lack thereof, of PB1-F2 protein was confirmed by confocal microscopy as described previously [14], [50] (data not shown).

### Live-cell bioimaging

NLRP3-deficient immortalized macrophages stably expressing ASC-cerulean and NLRP3 were seeded in 35 mm glass bottom culture dishes for 24 h prior to stimulation. Imaging was performed on a Leica SP5 multi-channel AOBS confocal laser scanning microscope equipped with a temperature and CO_2_-controlled sample chamber for live-cell imaging. PB1-F2 engulfment was imaged as deconvolved z-stacks by overlapping tile-scanning. Images were collected every 6 mins for a total of 2 or 3 h where indicated.

### Caspase-1 immunoblot

Immortalized macrophages (2×10^6^) were passaged in 6 well dishes 24 h prior to priming for 3 h with LPS (200 ng/ml) in serum free media. Macrophages were then stimulated for a further 6 h and cultured supernatants removed and centrifuged to remove cellular debris. Strataclean (Agilent) was added to supernatants, vortexed for 30 s and centrifuged (5 mins, 3000 rpm) to pellet matrix. Supernatants were aspirated and Laemmli buffer added to pellet, boiled and proteins separated by 4–14% gradient SDS-PAGE. Caspase-1 was imaged by immunoblot with anti-mouse Caspase-1 monoclonal antibody (Adipogen) and imaged by Licor Odyssey using anti-mouse-AlexaFluor 680.

### Animal model

Six- to eight- week old male or female C57BL/6 mice were maintained in the Specific Pathogen Free (SPF) Physical Containment Level 2 (PC2) Animal Research Facility at the Department of Microbiology and Immunology (University of Melbourne) and Monash Institute of Medical Research (Monash University). All experimental procedures were conducted under general anesthesia with inhaled isofluorane at 2.5% (Baxter Healthcare Corporation). Infectious agents and peptides were diluted in sterile PBS and administered intranasally to anaesthetized mice (n = 5/group). Mice were monitored daily for illness and morbidity. The infectious dose for all experiments was 100 Plaque Forming Units (PFU), which caused 10–15% weight loss and no mortality. NLRP3-deficient mice were kindly provided by Millennium Pharmaceuticals.

### BAL-F for cellular and cytokine assessment

Following euthanasia by CO_2_ inhalation, the trachea was exposed and BAL-F extracted as previously described [12]. Briefly, the proportion of neutrophils (Ly6G^+^, F480^−^ within the cellular region), macrophages (CD11c^+^, MHC class II^int^ within the cellular region) and DCs (CD11c^+^MHC class II^high^, within the cellular region) were assessed as a proportion of cellular events analyzed by flow cytometry as related to the number of white blood cells per mL (WBC/mL) determined by haematocrit examination using the Trypan Blue exclusion method. Cytokines in BAL-F were assayed as described above.

### Statistical analysis

Statistical analyses were performed using GraphPad Prism Version 5.

## Supporting Information

Figure S1 **PR8 PB1-F2 enhances cellular infiltration and IL-1β secretion in the lung.** Groups of mice (n = 5, 6–8 weeks, C57BL/6) were intranasally infected with 100 PFU X31 or ΔPB1-F2/X31 virus or treated with 5 µg PR8 or Wuhan peptide, or PBS. Figure shows **A**) cellular content of the BAL-F examined by flow cytometry for neutrophils, macrophages and dendritic cells (DCs); **B**) IL-1β secreted into the BAL-F determined by ELISA; **C**) IL-1β mRNA expression by RT-PCR 48 hpi (see also supplemental methods); **D**) cellular content of the BAL-F was examined by flow cytometry for neutrophils, macrophages and DCs; **E**) IL-1β secreted into the BAL-F determined by ELISA; *** p<0.001, ** p<0.01, * p<0 .05 compared to Wuhan, Δ p<0.001 compared to all groups, one way ANOVA, Tukey post-hoc. ΦΦ p<0.01 and Φ p<0.05 Student's unpaired T-test. Data is representative of two independent experiments and are represented as mean ± SEM.
(TIF)

Figure S2 **High molecular weight aggregated PB1-F2-induces ASC speck formation.** NLRP3-deficient macrophages reconstituted with ASC-cerulean (blue) and NLRP3 were seeded in 8 chamber optical chamber dishes (Ibidi) 24 h prior to stimulation with pHrodo labeled PB1-F2 peptide that has been separated into high (>100 kDa) and low (<100 kDa) molecular weight fractions by size exclusion. Cells were treated with peptide for 0, 30, 60 and 90 mins, fixed and visualized in 3D by z-stack collection in Imaris. 30 and 60 mins data are shown. Data is representative of two independent experiments.
(TIFF)

Figure S3 **PR8 PB1-F2 cannot induce IL-1β secretion in the absence of NLRP3.** Peritoneal macrophages (2×10^5^/mL) obtained from wild type and NLRP3-deficient mice, were primed with LPS, then exposed to PR8 PB1-F2 (5–50 µg/mL), silica (125 µg/mL), nigericin (10 µM), or the AIM2 activator poly (dA∶dT) (250 ng/mL) for a further 6 h. Cellular supernatants were collected and analyzed for IL-1β secretion by ELISA according to manufacturer's instructions. Results are representative of three independent experiments and are represented as mean ± SEM. * p<0.05, ** p<0.01 compared to NS (non-stimulated), ANOVA Dunnett's Multiple Comparison Test.
(TIF)

Text S1 **Supplementary methods: Quantitative IL-1β mRNA detection using PCR.**
(DOCX)

Video S1 **PR8 PB1-F2 peptide induces ASC speck formation.** NLRP3-deficient macrophages reconstituted with ASC-cerulean (blue) and NLRP3 were cultured in optical culture dishes (Ibidi) and treated with pHrodo-labeled (red) PB1-F2 peptide and visualized in 3D by z-stack collection (6 confocal planes) every 6 mins for 3.5 hours. Rapid phagocytosis of PB1-F2 peptide induces increased fluorescence of labeled peptide due to decreased pH of phagolysosomal compartment. Uptake of PB1-F2 peptide induces oligomerization of ASC in cytosol to form multimeric ASC speck. Movie is a maximum intensity projection of 3D image series from Imaris. Movie is representative of three independent experiments.
(MOV)

Video S2 **PB1-F2 peptide induces ASC speck formation across multiple cells.** NLRP3-deficient macrophages reconstituted with ASC-cerulean (blue) and NLRP3 were cultured in optical culture dishes (Ibidi) and treated with pHrodo-labeled (red) PB1-F2 peptide and visualized in 3D by z-stack collection (6 confocal planes) every 6 mins for 3.5 hours. Movie is a maximum intensity projection of 3D image series from Imaris. Movie is representative of three independent experiments.
(MOV)

Video S3 **Inhibition of phagocytosis blocks PB1-F2-induced ASC speck formation.** NLRP3-deficient macrophages reconstituted with ASC-cerulean (blue) and NLRP3 were pre-cultured in optical culture dishes (Ibidi) and treated with 20 µM of Latrunculin A for 40 mins prior to stimulation with pHrodo-labeled (red) PB1-F2 peptide. Cells were visualized in 3D by z-stack collection (6 confocal planes) every 6 mins for 90 mins. Movie is a maximum intensity projection of 3D image series from Imaris. Movie is representative of two independent experiments.
(WMV)
