# Supplementary figures and images for: Activation of the NLRP3 Inflammasome by IAV Virulence Protein PB1-F2 Contributes to Severe Pathophysiology and Disease
Source: PLoS Pathog. 2013 May 30;9(5):e1003392. doi: 10.1371/journal.ppat.1003392 (PMC3667782; doi:10.1371/journal.ppat.1003392)

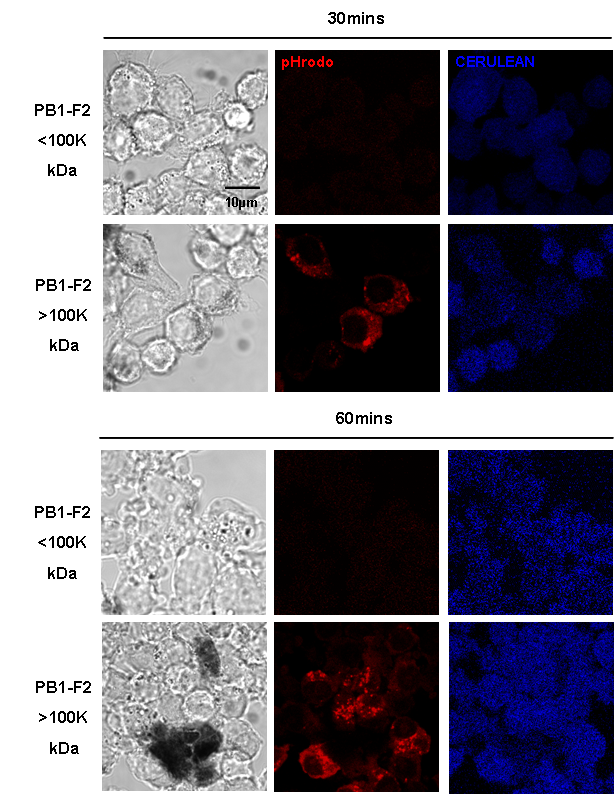

Supplement: Figure S2 — High molecular weight aggregated PB1-F2-induces ASC speck formation. NLRP3-deficient macrophages reconstituted with ASC-cerulean (blue) and NLRP3 were seeded in 8 chamber optical chamber dishes (Ibidi) 24 h prior to stimulation with pHrodo labeled PB1-F2 peptide that has been separated into high (>100 kDa) and low (<100 kDa) molecular weight fractions by size exclusion. Cells were treated with peptide for 0, 30, 60 and 90 mins, fixed and visualized in 3D by z-stack collection in Imaris. 30 and 60 mins data are shown. Data is representative of two independent experiments. (TIFF) [file ppat.1003392.s002.tiff]

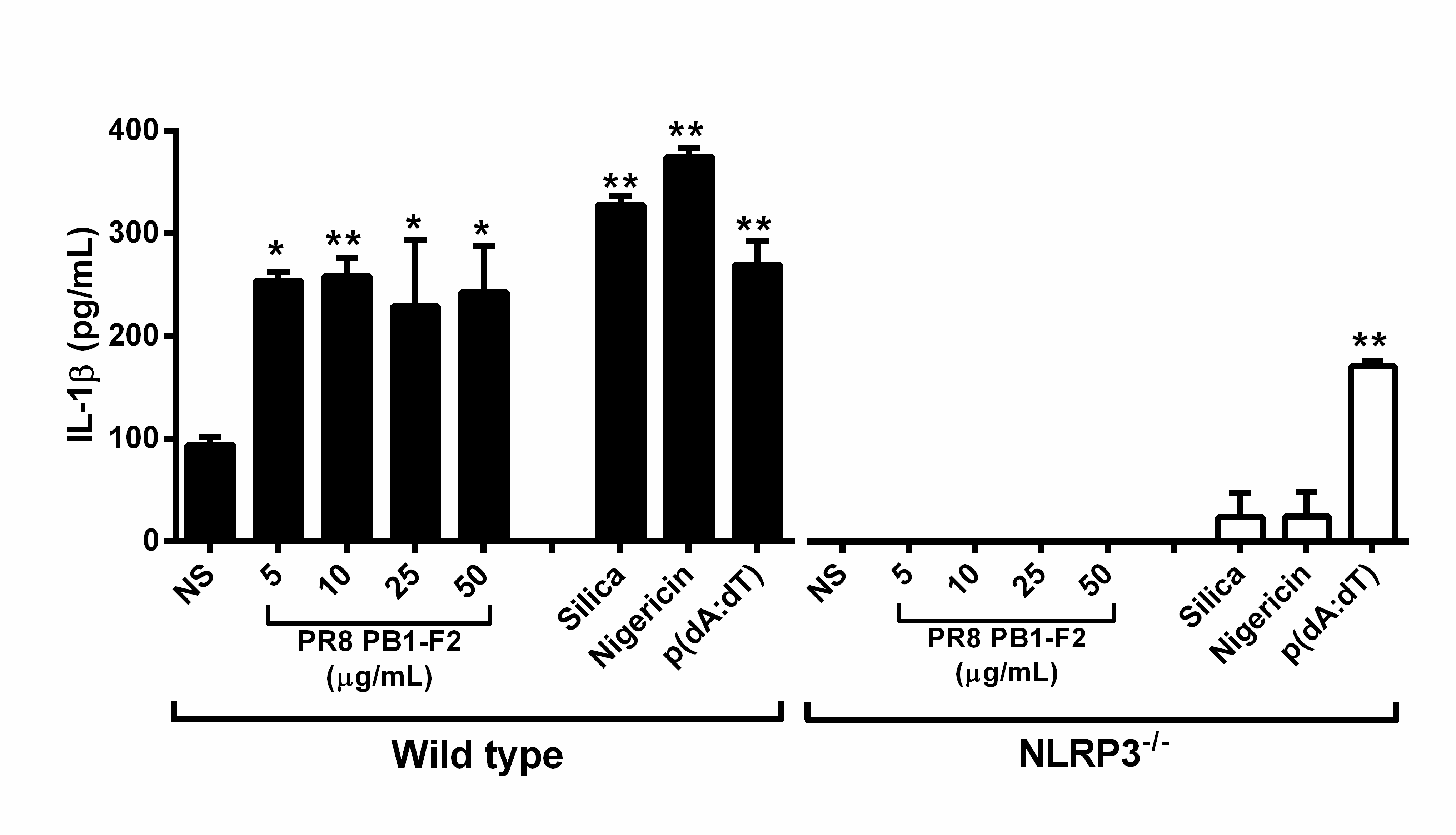

Supplement: Figure S3 — PR8 PB1-F2 cannot induce IL-1β secretion in the absence of NLRP3. Peritoneal macrophages (2×10^5^/mL) obtained from wild type and NLRP3-deficient mice, were primed with LPS, then exposed to PR8 PB1-F2 (5–50 µg/mL), silica (125 µg/mL), nigericin (10 µM), or the AIM2 activator poly (dA∶dT) (250 ng/mL) for a further 6 h. Cellular supernatants were collected and analyzed for IL-1β secretion by ELISA according to manufacturer's instructions. Results are representative of three independent experiments and are represented as mean ± SEM. * p<0.05, ** p<0.01 compared to NS (non-stimulated), ANOVA Dunnett's Multiple Comparison Test. (TIF) [file ppat.1003392.s003.tif]
