# Supplementary material for: Activation of the NLRP3 Inflammasome by IAV Virulence Protein PB1-F2 Contributes to Severe Pathophysiology and Disease
Source: PLoS Pathog. 2013 May 30;9(5):e1003392. doi: 10.1371/journal.ppat.1003392 (PMC3667782; doi:10.1371/journal.ppat.1003392)
Supplement: Text S1 — Supplementary methods: Quantitative IL-1β mRNA detection using PCR. (DOCX) [file ppat.1003392.s004.docx]

**SUPPLEMENTAL METHODS**

**Reverse Transcription-PCR Analysis**

WT mice were challenged with PBS, X31 and X31ΔPB1-F2 virus for 24 h as previously described. Lungs for infected mice were harvested and 100mg of lung tissue homogenized in Trisure. Total cellular RNA was prepared using the RNeasy mini kit according to the manufacturer's instructions (Qiagen). One μg of total RNA was treated with DNase (Promega) before cDNA synthesis using Superscript III (Life Technologies) and random primers in a volume of 40 μL. cDNA was analyzed in triplicate wells for each sample on the 7900HT Fast Real-Time PCR system (ABI, Foster City, CA). Samples were labeled with SyberGreen as per manufacturer’s instructions. Relative gene expression was determined using the cycling threshold method (2^-ΔΔCT^) and values normalized to GAPDH. The SyberGreen assays used were: mouse IL-1β (F: CAACCAACAAGTGATATTCTCCATG R: GATCCACACTCTCCAGCTGCA) and mouse GAPDH (F: CATGGCCTTCCGTGTTCCTA, R: GCGGCACGTCAGATCCA).
